# Measuring DNA content in live cells by fluorescence microscopy

**DOI:** 10.1186/s13008-018-0039-z

**Published:** 2018-09-04

**Authors:** Cecil J. Gomes, Michael W. Harman, Sara M. Centuori, Charles W. Wolgemuth, Jesse D. Martinez

**Affiliations:** 1 University of Arizona Cancer Center, University of Arizona, 1515 N. Campbell Ave, Tucson, AZ 85724 USA; 2 Department of Cell & Molecular Medicine, University of Arizona, Tucson, AZ 85724 USA; 3 Department of Physics, University of Arizona, Tucson, AZ 85724 USA; 4 Department of Molecular & Cellular Biology, University of Arizona, Tucson, AZ 85724 USA; 5 Cancer Biology Graduate Interdisciplinary Program, University of Arizona, Tucson, AZ 85724 USA; 6 Rhode Island Hospital, Providence, RI 02903 USA; 7 Department of Engineering, Brown University, Providence, RI 02912 USA

**Keywords:** Live-cell microscopy, Hoechst 33342, Imaging, DNA content

## Abstract

**Background:**

Live-cell fluorescence microscopy (LCFM) is a powerful tool used to investigate cellular dynamics in real time. However, the capacity to simultaneously measure DNA content in cells being tracked over time remains challenged by dye-associated toxicities. The ability to measure DNA content in single cells by means of LCFM would allow cellular stage and ploidy to be coupled with a variety of imaging directed analyses. Here we describe a widely applicable nontoxic approach for measuring DNA content in live cells by fluorescence microscopy. This method relies on introducing a live-cell membrane-permeant DNA fluorophore, such as Hoechst 33342, into the culture medium of cells at the end of any live-cell imaging experiment and measuring each cell’s integrated nuclear fluorescence to quantify DNA content. Importantly, our method overcomes the toxicity and induction of DNA damage typically caused by live-cell dyes through strategic timing of adding the dye to the cultures; allowing unperturbed cells to be imaged for any interval of time before quantifying their DNA content. We assess the performance of our method empirically and discuss adaptations that can be implemented using this technique.

**Results:**

Presented in conjunction with cells expressing a histone 2B-GFP fusion protein (H2B-GFP), we demonstrated how this method enabled chromosomal segregation errors to be tracked in cells as they progressed through cellular division that were later identified as either diploid or polyploid. We also describe and provide an automated Matlab-derived algorithm that measures the integrated nuclear fluorescence in each cell and subsequently plots these measurements into a cell cycle histogram for each frame imaged. The algorithm’s accurate assessment of DNA content was validated by parallel flow cytometric studies.

**Conclusions:**

This method allows the examination of single-cell dynamics to be correlated with cellular stage and ploidy in a high-throughput fashion. The approach is suitable for any standard epifluorescence microscope equipped with a stable illumination source and either a stage-top incubator or an enclosed live-cell incubation chamber. Collectively, we anticipate that this method will allow high-resolution microscopic analysis of cellular processes involving cell cycle progression, such as checkpoint activation, DNA replication, and cellular division.

**Electronic supplementary material:**

The online version of this article (10.1186/s13008-018-0039-z) contains supplementary material, which is available to authorized users.

## Background

In biological sciences, the most universally measured genomic constituent is DNA content. Its quantification serves to assess several cellular parameters including DNA ploidy and a cell’s temporal location within the cell cycle [1]. DNA content is accurately assessed on a single cell basis by measuring the integrated nuclear fluorescence of a fluorophore that binds to DNA stoichiometrically. Traditionally, reporting DNA content has been accomplished with a high degree of accuracy in large cell populations using flow cytometry, a technique that allows the proportion of cells in each phase of the cell cycle to be calculated in a high-throughput fashion. However, the measurement of DNA content by flow cytometry provides little resolution to the biology of individual cells. To overcome this limitation, approaches incorporating laser scanning cytometry and fluorescence microscopy have been successful in quantifying DNA content in single cells [2, 3], providing the capacity to combine this information with additional cellular parameters that can be elucidated by imaging.

Many of the fluorophores that bind to DNA stoichiometrically are incompatible with live cells; the difficulty lies in granting the DNA fluorophore—often not membrane-permeant as with DAPI, PI, and 7-AAD—access to the DNA [4, 5]. Therefore, traditional methods for examining DNA content by microscopy have relied on cellular fixation, which is incompatible with tracking cells over time. To overcome the limitations brought upon by cellular fixation, membrane-permeant DNA fluorophores were designed to stain DNA stoichiometrically in live-cells (supravital staining), enabling both the quantification of DNA content and the ability to track the movement of chromosomes over time. However, it quickly became evident that exposure to membrane-permeant DNA fluorophores, such as Hoechst 33342 and DRAQ5, induce distinct DNA damage responses in cells, including activation of ATM, Chk2 and p53 [6]. Furthermore, repeated exposure to UV light is particularly damaging to cells as it causes photolysis of DNA [7]. Consequentially, the cytotoxicity and phototoxicity associated with the use of supravital dyes often result in cell cycle arrest and apoptosis [8, 9]. Therefore, these dyes have limited use in long-term LCFM applications and should be employed sensibly in acute studies examining cell cycle progression or apoptosis as their cytotoxic effects cannot be ignored. These limitations have made quantifying DNA content in LCFM applications challenging, yet this capacity would allow for cell cycle staging and DNA content to be coupled with any variety of cellular dynamics obtained by LCFM.

Here we attempt to bridge that void and present a widely applicable nontoxic procedure for measuring DNA content in live-cells at the end of a LCFM experiment, allowing individual cells to be imaged for any interval of time followed by the quantitation of their DNA contents. This capacity allows for high-resolution microscopic analysis of cellular processes involving cell cycle progression. This approach is compatible with LCFM applications because it avoids the toxicity typically associated with long-term exposure to live-cell DNA dyes. Furthermore, a variety of live-cell DNA fluorophores can be utilized; here we present this procedure using Hoechst 33342, a live-cell DNA dye that binds to AT-rich sequences in the minor groove of double-stranded DNA, allowing for a stoichiometric relationship between the amount of DNA present and Hoechst fluorescence [10].

We also demonstrate how this method, when used in combination with a histone 2B-GFP fusion protein, can be employed to monitor chromosomal dynamics in cells of varying ploidies (2n, 4n, 8n); an approach we recently used to examine the length of mitosis and the frequency of mitotic errors in polyploid cells induced by the overexpression of YWHAG [11]. To that end, cells with polyploid DNA content have recently been demonstrated to facilitate rapid adaptation in human tumors through significantly elevated rates of genomic aberrations, ranging from single nucleotide changes to whole chromosome gains and losses [12]. These aberrations, collectively termed genetic instability, are characteristic to human cancers [13] and is the primary source for genetic variability that selects for populations with increased malignancy and resistance to therapy [14]. These observations have fueled the notion that polyploid cells exist as unstable intermediates en route to aneuploidy [15–17]. In addition to allowing for the characterization of cellular stage by LCFM, the procedure presented here enables the enumeration of chromosomal segregation errors in diploid and polyploid cell populations in high-resolution.

## Methods

### Cell culture and transfections

The model systems for the cell culture are NCI-H322 (ATCC, Manassas, VA) human bronchioalveolar carcinoma cells, which were maintained in Dulbecco’s modified Eagle’s medium (Corning Cellgrow, Manassas, VA, USA) supplemented with 10% fetal bovine serum (Peak Serum, Fort Collins, CO), 100 U penicillin and 100 mg streptomycin (ThermoFisher, Waltham, MA, USA) and maintained at 37 °C in a humidified atmosphere of 5% CO_2_. Cells were transfected using Lipofectamine LTX with the addition of the manufacture’s PLUS reagent (Invitrogen, Carlsbad, CA, USA). Cells transfected with pBOS-H2B-GFP were selected with 3 μg/mL Blasticidin S HCl (Sigma-Aldrich, St. Louis, MO, USA), FACS sorted based on positive GFP expression, and maintained in 1 μg/mL Blasticidin S HCl (ThermoFisher, Waltham, MA, USA) within the culture medium. Cells transfected with pCMV-Tag2B-14-3-3γ were maintained in 400 μg/mL G418 (ThermoFisher, Waltham, MA, USA).

### Cell cycle profiling by flow cytometric analysis

Cells were fixed by drop-wise addition of 70% ice-cold ethanol while vortexing. Samples were then treated with RNase A (Sigma-Aldrich, St. Louis, MO, USA), stained with Propidium iodide (Sigma-Aldrich, St. Louis, MO, USA) and incubated at 37 °C for 30 min prior to cytometric analysis. Assessment of DNA content was carried out using a BD FACScanto II flow cytometer (BD Biosciences, San Jose, CA, USA) and cell cycle histograms generated using the FlowJo V10 (Ashland, Oregon) software package. Cell aggregates were gated out of the analysis, determined by PI-A and PI-W.

### Procedural setup

**Fig. 1:**
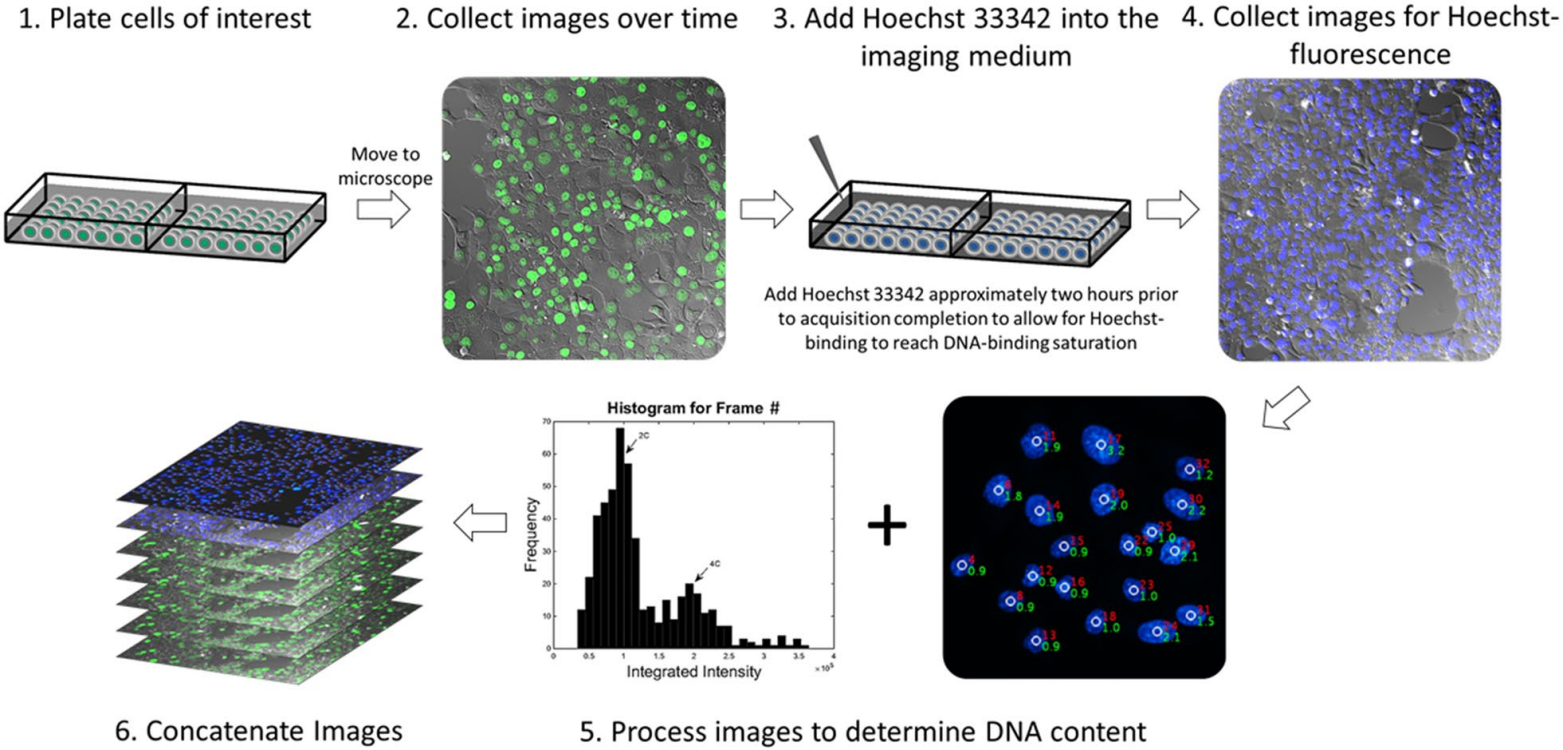
Procedural schematic for measuring DNA content in live cells. Cells of interest are plated in coverglass-bottom chambered slides and are later transferred to an inverted microscope for the collection of time-lapse images. The acquisition is then paused ~ 2 h before the completion of the time-lapse experiment and Hoechst 33342 is added to the imaging medium at a concentration of 1 μg/mL, the acquisition is then resumed. At the completion of the time-lapse experiment, images are collected for Hoechst 33342 fluorescence and analyzed with the ProcessDNA algorithm. The time-lapse images are then concatenated with the analyzed images for DNA content (steps 1–6)

Live-cell fluorescence microscopy comes with several challenges that must be addressed before this technique can be utilized (as reviewed in [18]). A schematic of the procedure is shown in Fig. 1 and consists of six major steps. (1) Cells were plated into 2-well coverglass bottom chambered slides (Thermofisher Scientific, Waltham, MA, USA) at a concentration of 20,000 cells/well and allowed to adhere for a minimum of 24 h at 37 °C and 5% CO_2_. Prior to the imaging acquisition, cells were washed with phosphate buffer saline (PBS) and the replenished with FluoroBrite DMEM imaging medium (ThermoFisher) supplemented with 10% Fetal Bovine Serum (Peak Serum) and 1% Penicillin–Streptomycin (Gibco). Cell synchronization may be performed to maximize the mitotic fraction of cells for studies aimed at investigating cell division dynamics in a high-throughput fashion. The chambered slide was then transferred to a Pecon Heating Insert (Carl Zeiss) attached to the microscope stage and maintained at 37 °C and 5% CO_2_. To ensure that the cells remain in focus for the duration of the imaging acquisition, the slide must be tightly secured to the stage-top incubator. This is a critical step in guaranteeing that the slide will not move in the latter half of the experiment while the supravital dye is added. (2) Time-lapse images were acquired using a 20× air objective with a CMOS camera (Orca Flash V4.0, Hamamatsu Photonics, Hamamatsu City, Japan) on a Zeiss AxioObserver.Z1 wide-field epifluorescence microscope equipped with an automated stage and focus (Carl Zeiss, Oberkochen, Germany). Differential interference contrast and fluorescence images were collected at 3-min intervals for 18 h at several regions of interest. (3) Approximately 2 h prior to the completion of the time-lapse experiment, the imaging acquisition was paused and the lever arm of the microscope raised, and the lid to the Pecon heating insert carefully removed. Hoechst 33342 (Thermo Fisher Scientific, Waltham, MA, USA) was then carefully added in a drop-wise fashion to the imaging medium without perturbing the slide position to a final concentration of 1 μg/mL. The lid to the heating insert and the lever arm were then re-positioned with similar care and image acquisition was resumed. Images were collected for an additional 2 h to allow for stoichiometric binding of Hoechst 33342. During this 2-h window, images were solely collected to ensure that single cells could be tracked throughout the duration of the time lapse experiment; these images were not assessed for cellular dynamics, as exposure to Hoechst 33342 has been demonstrated to cause cytotoxic effects [6, 8, 9]. (4) Upon reaching dye saturation, cells were then imaged for Hoechst 33342 fluorescence and all images collected were saved as uncompressed.avi files. This method is contingent on the precise measurement of DNA content emitted from Hoechst’s fluorescence, and therefore it is essential that the images that are collected not be saturated. (5) The integrated nuclear fluorescence of Hoechst 33342 was then calculated for each cell within a given frame and plotted to a cell cycle histogram using a MATLAB (MathWorks, Natick, MA) algorithm that we developed. The algorithm generates an additional.avi file with the DNA contents overlaid to the original Hoechst 33342 images. (6) Using ImageJ (National Institutes of Health), individual.avi stacks collected from the time-lapse experiment were concatenated with their respective images collected for Hoechst 33342 fluorescence and saved as contiguous.avi video files. Notably, Microscope setups furnished with an automated stage for imaging multiple fields of view, such as ours, requires that Ho342 is added to the imaging medium without moving the slide position, as even the slightest adjustment in its position can relinquish the existing focus strategy. This becomes less important when imaging a single field of view, as that location can be re-adjusted manually with ease.

### Image processing

Images of Hoechst 33342 fluorescence were analyzed using the ProcessDNA algorithm constructed to measure the integrated nuclear fluorescence of each cell. The algorithm computes the perimeter shape and the location of the center of mass of each nucleus from fluorescent images by combining a thresholded image and a moving least-squares algorithm, as previously described in Harman et al. [19]. The threshold value is determined based on the sharpest gradients in the image intensity. The integrated fluorescence is then equated over the area of each nucleus, and that data is exported into a histogram distribution, with the integrated intensities reduced to a two-digit read-out and binned accordingly based on the data distribution. This algorithm is simple in design and is constructed to rapidly assess 2D-images and allows users to determine where an individual cell resides within the cell cycle without having to use several software. Steps to use this pipeline and the code generated to run the ProcessDNA algorithm are provided (Additional files 1, 2).

## Results

### Hoechst 33342 binding saturation

**Fig. 2:**
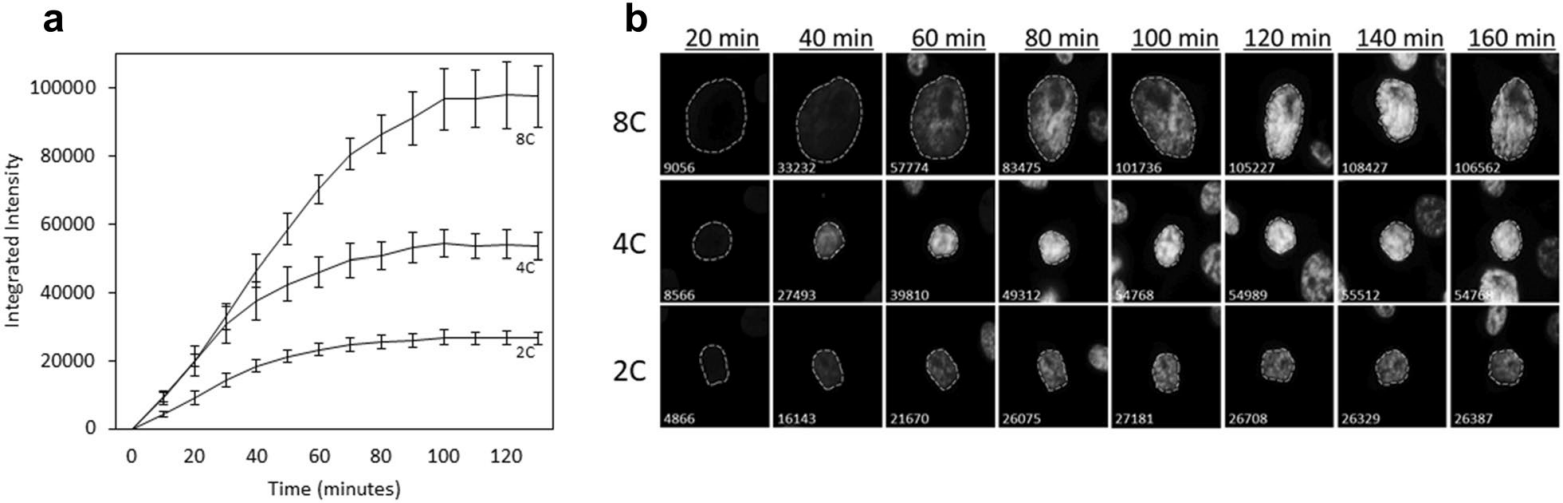
Determining the length of supravital dye saturation. Asynchronous cells were plated onto 8-well chambered slides and allowed 24 h to adhere. After the addition of Hoechst 33342 into the culture medium, fluorescent images were taken at 20-min intervals. **a** The integrated fluorescent intensity of cells with approximately 2C, 4C, and 8C DNA content are graphed over time, with error bars representing the standard deviation within groups. **b** Representative examples of cells with varying amounts of DNA content are presented in a time series with 20-min intervals. Located at the bottom left of each image are the integrated fluorescent units calculated at the corresponding time-point

The time it takes for Hoechst-binding to reach saturation must be determined empirically as the rate of dye-uptake is cell-type and concentration-dependent [20, 21]. Figure 2 illustrates the temporal span for Hoechst 33342 to reach binding saturation in H322 cells harboring different DNA contents at a concentration of 1 μg/mL. Following the dye’s addition, the integrated nuclear fluorescence of Ho342 was manually tracked in 50 cells over the course of 3 h with 10-min acquisition intervals. The integrated intensities collected from cells with DNA contents ranging from 2C to 4C became stable within approximately 100 min after the dye’s addition, indicating that Ho342 binding reached saturation (Fig. 2a). Cells with ~ 8C DNA content required an additional 20 min for the integrated intensities to stabilize. Markedly, diploid cells cycle between 2C (G1) and 4C (G2/M) DNA content, while tetraploid cells cycle between 4C (G1) and 8C (G2/M), therefore, 8C cells were categorized as polyploid cells.

A closer look at the integrated intensities over time showed marginal changes in fluorescence following the dye’s saturation, with greater fluctuations occurring in cells with inherently more DNA content (Fig. 2b). These slight fluctuations are characteristic to measuring Ho342-associated fluorescence in live cells, as they actively efflux Ho342 to varying degrees depending on the cell type [21]. The efflux of Ho342 in H322 cells appeared insignificant and did not require intervention; however, the co-incubation of Hoechst 33342 with efflux inhibitors such as Verapamil and trifluoperazine [22], or the membrane potential modifying fluorochrome DiOC5(3), is occasionally required to yield an accurate resolution of DNA content in cell-types with poor dye retention [22–24]. This data demonstrates that our approach is suitable for measuring DNA contents with a relatively good degree of accuracy, but that without efflux inhibitors, it should not be employed when attempting to measure minute numerical changes in chromosome complements in live cells.

### Staging live cells

**Fig. 3:**
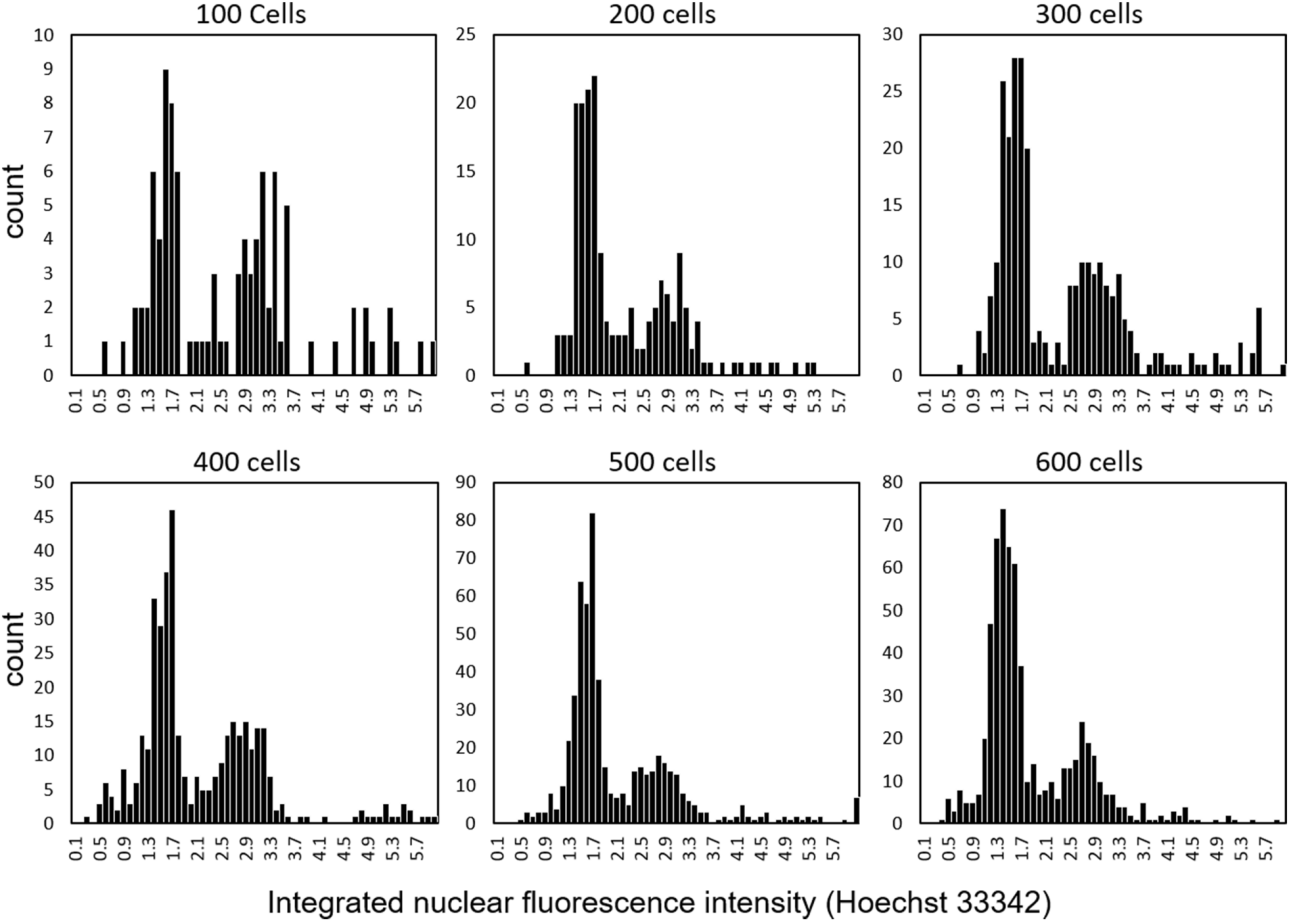
Cell cycle profiles obtained with varying cellular densities. Asynchronous cells were plated into 8-well coverglass-bottom chambered slides at varying densities and allowed to adhere for 24 h. Hoechst 33342 was added to the imaging medium and allowed to reach binding saturation (~ 2 h). Images for Hoechst 33342 were then collected at distinct locations and histograms generated from images containing approximately 100, 200, 300, 400, 500 and 600 cells within a single field of view. The x-axis represents normalized integrated nuclear fluorescence for each cell imaged and the y-axis histogram counts

To demonstrate the high-throughput approach of this methodology, we assessed the number of cells required within a single field of view (FOV) to generate a cell cycle profile with distinct separation between cell cycle phases. We treated live cells with Ho342 until binding reached saturation and then captured profiles at densities of ~ 100–600 cells per FOV. Notably, similar cell counts are obtainable at lower cellular densities by imaging multiple fields of view and combining that data into a single histogram. Cell cycle phases G1, S, and G2/M, were defined by applying manual gates to the cell cycle histogram. We observed distinct populations of all three phases when a minimum of 200 cells were assessed within a single field of view, but more discernable cell cycle profiles emerged as cell counts increased (Fig. 3). Next, we investigated whether cell cycle profiles collected from live cells stained with Ho342 could recapitulate data collected from flow cytometry. Therefore, we performed a side-by-side experiment comparing asynchronous cell cycle distributions acquired in live cells by fluorescence microscopy to that of fixed cells acquired by flow cytometry. The analysis of 10,000 cells by flow cytometry yielded a distribution of 60.7% in G1, 17.0% in S, and 20.5% in G2/M (Additional file 3: Figure S1). Several fluorescent images were collected in live cells plated at different densities with a 20× 0.8-N.A. lens, and cell cycle profiles were assessed with manual gate placement (Table 1). When all three phases were examined together, images containing 500–699 cells within a field of view were most accurate at resolving cell cycle distributions similar to what was observed by flow cytometry. Notably, at all densities, the proportion of cells in each phase were lower than that acquired by flow cytometry, with noticeably underrepresented proportions of cells in S phase. This is likely due, in part, to the conservative placement of each gate; which were positioned cautiously because of the wide-ranging profiles of G1 and G2/M generated by imaging as opposed to the narrower spread observed by flow cytometry (Additional file 3: Figure S1b). Furthermore, doublets were gated out of the cell cycle profile acquired by flow cytometry, whereas nuclei that are positioned closely with one another that also lie within the accepted size threshold are occasionally characterized as a single nucleus with the ProcessDNA algorithm. This occurrence can be seen in cells profiled to the right of the G2/M population (Additional file 3: Figure S1b), and therefore reduce the proportion of cells that lie within the G1, S, and G2/M gates. Collectively, we conclude that our method can be used to stage individual cells in the cell cycle with a relatively good degree of accuracy when data for a minimum of 500 cells are collected.

**Table 1:** Cell cycle distributions from images with varying densities of cells

Cells per image	Total number of images assessed	G1^a^ (% ± S.D)	S^a^ (% ± S.D)	G2/M^a^ (% ± S.D)
100–299	6	46.1 ± 6.6	10.4 ± 3.8	17 ± 3.2
300–499	5	49.7 ± 5.1	9.90 ± 2.3	18.5 ± 2.8
500–699	4	58.1 ± 2.4	10.4 ± 1.3	15.2 ± 0.3

^a^Percentage of cells in each stage of the cell cycle ± standard deviation (S.D)

### Examining polyploid cell division

Studying the molecular mechanisms that affect cellular division is crucial to our understanding of genomic stability. When this process fails to occur faithfully, daughter cells may inherit abnormal chromosome complements with structural and numerical aberrations—characterized as aneuploidy. Because cells with abnormally elevated levels of DNA content have inherently increased rates of genomic instability and are widely considered drivers of aneuploidy [15–17], we reasoned that our approach of measuring DNA content in live cells could be coupled with the constitutive expression of H2B-GFP to study mitotic aberrations in polyploid cells. Importantly, the use of fluorescently labeled histones is compatible with long-term live-cell imaging applications and has been used in several studies investigating chromosomal segregation errors, such as lagging chromosomes, multipolar mitoses, and anaphase bridges [25–27].

To asses this, we first employed a cell line harboring diploid and polyploid populations established by overexpression of the *YWHAG* oncogene [28]. We then introduced the constitutive expression of H2B-GFP into these cells to allow for the spatiotemporal movement of mitotic chromosomes to be visualized in high-resolution. LCFM was performed with images collected in 3-min intervals for 18 h and the DNA contents of each cell calculated at the end of the experiment as described within. Cell cycle profiles were generated and referenced to define 2C, 4C, and 8C populations. The ploidy of dividing cells, that is, the number of complete sets of chromosomes, were successfully calculated by summing together the DNA contents in emerged daughter cells, i.e., diploid cells tracked through mitosis were identified by the DNA contents of their daughter cells adding up to 4C, and tetraploid cells similarly identified by the summation of 8C (Additional file 4: Figure S2). Notably, the precise characterization of cellular ploidy in mitotic cells was contingent on measuring DNA content in the daughter cells while they remained in growth phase I of the cell cycle. Concatenation of all images collected resulted in a time-lapse video that allowed for cellular ploidy and mitotic progression to be assessed contiguously (Additional file 5: Video S1).

**Fig. 4:**
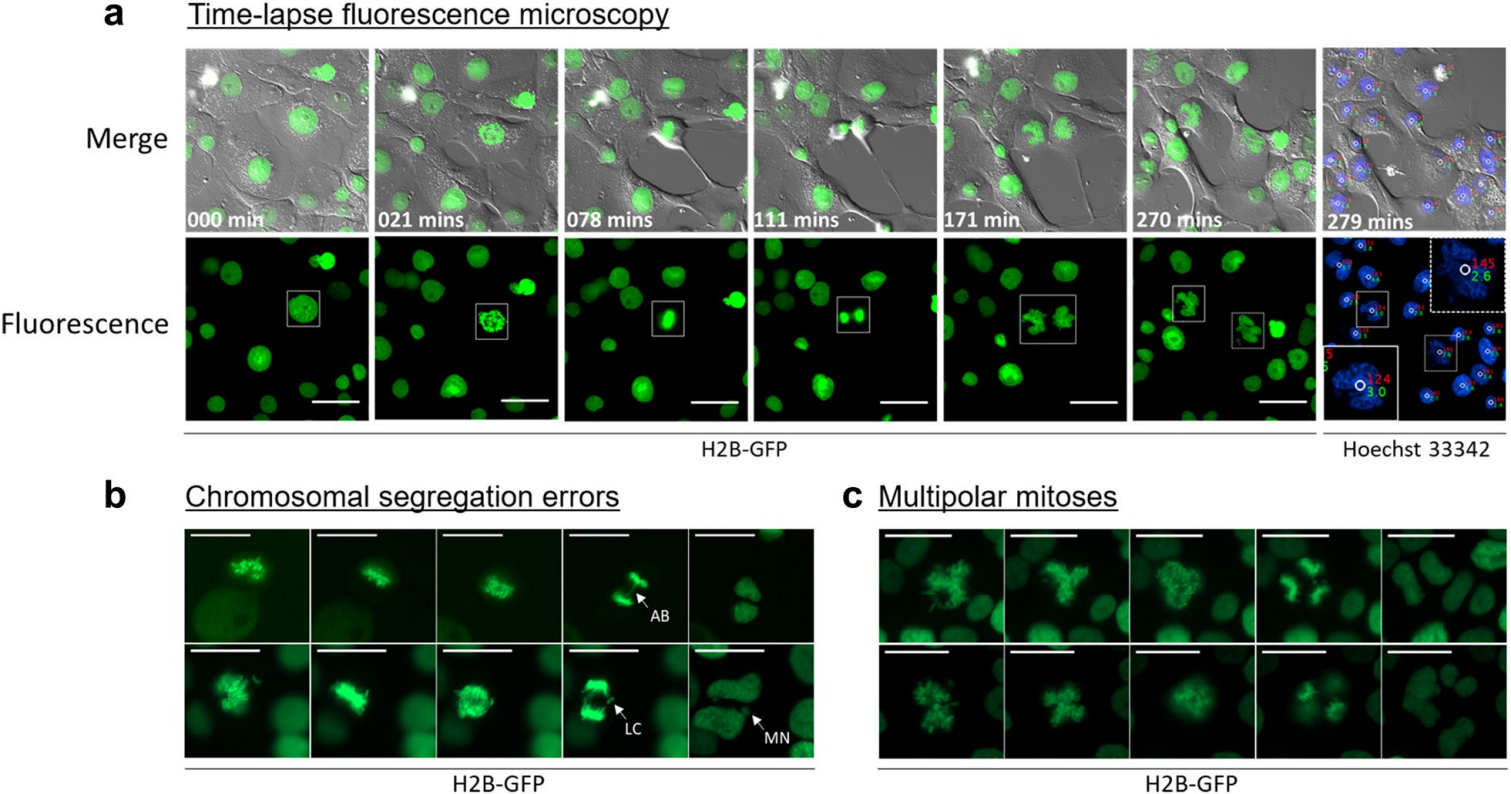
Polyploid cells occasionally experience error prone mitoses. H2B-GFP labeled cells were imaged over a 20-h time course with 3-min intervals between acquisitions. At the 18-h mark, Hoechst 33342 was added to the imaging medium. At the completion of the time-lapse experiment, images were collected for Hoechst 33342 fluorescence. Hoechst 33342 images were analyzed using the ProcessDNA pipeline and concatenated to the time-lapse series. **a** Highlighted is a polyploid cell progressing through mitosis with asymmetrical separation of DNA between daughter cells. Scale bar = 20 μm. **b** Mitotic errors such as anaphase bridges (AB, top panel) and lagging chromosomes (LC, bottom panel) with subsequent micronuclei production (MN) were observed. Scale bar = 10 μm. **c** Asymmetrical separation of DNA occasionally resulted from tripolar (top panel) and quadripolar (bottom panel) spindles. Scale bar = 10 μm

Figure 4 illustrates several mitotic defects that can be examined using this system, such as asymmetrical separation of DNA (Fig. 4a), fragmented nuclear morphologies (Fig. 4a), anaphase bridges (Fig. 4b, top panel), lagging chromosomes with subsequent micronuclei formation (Fig. 4b, bottom panel) and multipolar mitoses (Fig. 4c). Collectively, the expression of H2B-GFP combined with the quantification of DNA content—as outlined in this protocol—enables DNA ploidy to be correlated with several cellular parameters, and was recently employed to examine the length of mitosis and to enumerate the frequency of mitotic errors in polyploid cells induced by overexpression of *YWHAG* [11].

## Discussion

We have established a universal approach for measuring DNA content in live cells that is compatible with long-term LCFM. By introducing Ho342 into the culture medium of H322 cells and allowing it to bind stoichiometrically, we were able to measure DNA content in individual cells and thus determine each cell’s stage within the cell cycle. The quantification of DNA content in individual cells was streamlined using the ProcessDNA algorithm, a Matlab-derived script that recognizes individual cells and measures the integrated nuclear fluorescence intensity of Ho342. This combined with time-lapse images collected for H2B-GFP allowed us to track individual cells and their DNA contents back through time, providing a history of the cell’s progression through the cell cycle. Importantly, our procedure avoids cell cycle perturbations and DNA damage caused by live cell dyes, such as Ho342, by limiting cellular exposure to a short period of time at the end of the experiment. Hence, we were able to observe chromosome dynamics associated with the cell cycle, unperturbed by Ho342 staining. Using this approach, we linked DNA content and cell cycle staging to individual cells tracked in a time-lapse fashion.

We were able to generate cell cycle distribution profiles with distinct populations of stages G1, S, and G2/M using image processing. We found that optimal resolution appeared when at least 500 cells were assessed for cell cycle distributions (Fig. 3). This number of cells was easily obtained by imaging a single FOV using a 20× 0.8-N.A. objective lens. Our method and the ProcessDNA algorithm are also compatible with the use of a 10× objective if cells are to be imaged at a lower cellular confluence. For studies seeking to acquire cell cycle profiles with counts similar to what is achieved by flow cytometry, the DNA contents from multiple fields of view can be compiled and DNA histograms can then be generated from this data with spreadsheet software such as Excel or statistical software R [29].

Generating accurate cell cycle profiles is dependent on the correct segmentation of nuclei for quantitating DNA content. The ProcessDNA algorithm was designed to detect individual nuclei on a flat 2D-plane. This can be difficult when analyzing images from cancer cells as they frequently grow in clusters and atop one another. Therefore, the plating density should be optimized for a sufficient number of cells within a single field of view, but not too dense such that cells are growing atop one another. To mitigate this phenomenon, the chambered slides used for imaging can be pre-coated with Poly-l Lysine or fibronectin, which can encourage cell adhesion to the underlying surface [30]. Nevertheless, nuclei detection and nuclear fluorescence measurements can also be performed using CellProfiler [31], an open-source image analysis pipeline that is suitable for detecting individual nuclei within cell clusters. Although timely, the assessment of DNA content can also be performed manually by integrating the nuclear fluorescence using software such as ImageJ. Alternatively, images can be collected at several z-planes (stacks) to account for the variability of cells in different z-positions. The stack of images can then be averaged for all z-positions and the integrated intensity of Ho342 can be calculated [29].

It was evident that cell cycle profiles generated by imaging, although similar, did not precisely mirror profiles generated by flow cytometry. Studies requiring precise cell cycle staging using this method should consider the use of efflux inhibitors alongside the addition of Ho342 into the imaging medium to preserve dye retention. This will reduce the lower-bound spread of G1 and G2/M populations, ultimately making all three phases more distinct. Our method alone appears sufficient for staging live cells within the cell cycle but is limited temporally to the end of the time-lapse experiment. To resolve cell cycle stages throughout the duration of the time-lapse experiment, the additional use of live cell cycle reporters can be utilized. The expression of fluorescently labeled proliferating cell nuclear antigen (PCNA) can be incorporated into the system to further clarify cells that are in S phase from those in G1 and G2/M [32]. Alternatively, the fluorescence ubiquitination cell cycle indicator (FUCCI) system can be used to identify cells in G1 from those in S/G2/M phases [33]. The integration of these cell cycle reporters may increase the accuracy of staging live cells, but do not provide resolution of all three phases and also requires the use of additional spectral imaging channels, reducing the capacity for visualizing of cellular features by fluorescence imaging and consequently should be employed pragmatically.

Our method of measuring DNA content at the end of a time-lapse experiment was recently performed to study polyploid cells as they progressed through cell division [11]. We reasoned that polyploid cells could be identified using our method, as DNA content and DNA ploidy are interdependent. Cells undergoing mitosis were visualized by differential interference microscopy. Thus, cells that emerged from mitosis were accurately staged in G1 of the cell cycle and the amount of DNA content directly related to their ploidy. With the concomitant expression of H2B-GFP, mitotic dynamics were assessed in cells that were retrospectively identified as diploid or polyploid. Our method is, therefore, an attractive new technique for exploring unresolved cell division and proliferation dynamics of polyploid cells.

## Conclusions

We have developed a LCFM technique that allows tracking of single cells through an unperturbed cell cycle for an extended period and the subsequent quantification of their DNA content by automated image analysis. We outline a protocol for a standard wide-field fluorescence microscope (e.g., AxioObserver.Z1, Carl Zeiss) equipped with a UV light source for the excitation of the DNA dye and a low-cost lab-standard 20× 0.8-NA air objective lens. This configuration allows the quantification of Hoechst-stained cells and the derivation of cell cycle profiles without the need for 3D image acquisition. This method is wide-ranging, as the use of alternative epifluorescence or confocal microscopes will allow similar results to be achieved. Furthermore, this approach is compatible with the use of any stiociometric live-cell DNA dye, permitting the occupancy of alternative fluorescent channels. Collectively, we anticipate that this method will allow high-resolution microscopic analysis of cellular processes involving cell cycle progression, such as checkpoint activation, DNA replication, and cellular division.

## Additional files

**Additional file 1.** Using the ProcessDNA algorithm for measuring DNA content.

**Additional file 2.** ProcessDNA MATLAB file.

**Additional file 3: Figure S1.** Side-by-side comparison of cell cycle profiles derived from flow cytometry and by live-cell fluorescence microscopy. Flow cytometry and fluorescence image analysis data was obtained from asynchronous cells that were plated in either 6-well culture dishes or 2-well chambered slides, respectively. a) The cell cycle profile displayed was generated from cells that were fixed and treated with propidium iodide and ribonuclease A. 10,000 events were collected with doublets gated out of the analysis by SCC and PI-A. Manual gates were placed to determine the percentage of cells within the different cell cycle phases shown. b) Displayed is a cell cycle profile generated from live-cell fluorescence microscopy of Hoechst 33342 stained cells (n = ~ 600 cells). The corresponding image is presented in the top-right corner to illustrate the plating density required for 600 cells per field of view.

**Additional file 4: Figure S2.** Calculating cellular ploidy in live cells using Hoechst 33342. A diagrammatical representation of H2B-GFP labeled cells progressing through mitosis (grey arrows) is shown. DNA ploidy can be calculated for each mitotic cell by summing the nuclear fluorescence of Hoechst 33342 in the nascent daughter cells. A diploid and tetraploid example is illustrated.

**Additional file 5: Video S1.** LCFM was performed on cells labeled with H2B-GFP (green fluorescence), and each cell’s DNA content was later measured using Hoechst 33342 staining (blue fluorescence), as described within. All images were then concatenated and the ProcessDNA algorithm was employed to quantify DNA content.
