# Supplementary material for: Measuring DNA content in live cells by fluorescence microscopy
Source: Cell Div. 2018 Sep 4;13:6. doi: 10.1186/s13008-018-0039-z (PMC6123973; doi:10.1186/s13008-018-0039-z)
Supplement: Supplementary file 1 — Additional file 1. Using the ProcessDNA algorithm for measuring DNA content. [file 13008_2018_39_MOESM1_ESM.docx]

**Additional file 1**

Using the ProcessDNA algorithm for measuring DNA content:

1. Before opening MATLAB, copy the ‘ProcessDNA.m’ file into the working directory of the .avi files that are going to be analyzed.
2. Launch MATLAB and navigate the working directory to the intended folder for analysis.
3. To run the algorithm, enter the following into the command window:

[DataSet] = ProcessDNA(‘moviename.avi’,ThreshValue,MinSize,MaxSize,PixelToMicronRatio,AviFlag)

Δ The threshold (ThreshValue) and size of the nuclei (MinSize, MaxSize) will have to be optimized accordingly. The threshold value is determined based on the sharpest gradients in the image intensity. Notably, any object smaller than the MinSize or larger than the MaxSize is removed from the segmented image.

For example: “[DataSet] = ProcessDNA(‘test.avi’,0.5,200,10000,.128,1)”, where ‘test’ is the .avi file name, 0.5 is the threshold value, 200 is the minimum size allowed to be considered a nucleus, 10000 is the maximum size allowed to be considered a nucleus, .128 is the pixel to micron conversion factor, and 1 is used to trigger the pipeline to recognize the .avi file. However, if a stack of images with a file type other than .avi are to be analyzed, use ‘0’ instead of ‘1’.

1. This pipeline measures the integrated nuclear intensities within a given image and generates a histogram plotting the data. Furthermore, a .avi video will automatically be generated from the tracked data and stored in the working directory named ‘tracked.filename’. The number for each nucleus analyzed is presented in red and the DNA content directly below that in green.

Construction of the ProcessDNA algorithm:

Using the MATLAB *VideoReader* command, an object named ‘MovieObj’ was created to read video data from a given .avi file. The number of frames within the MovieObj were designated by ‘MovieObj.NumberofFrames’. To extract the size of the image from the MovieObj, the height and width of each frame in pixels are given as ‘MovieObj.Height’ and ‘MovieObj.Wdith’, respectively. An objective, AviOut, was then named to write video data using *VideoWriter*, with an array of X, Y coordinates for the pixels in an image. Since Hoechst 33342 reached binding saturation approximately two hours following its addition to the imaging medium, only the last five frames of the Hoechst .avi movie (MovieObj) were designated to be read. This can be adjusted by altering the n value in line 34: for example, the last ten frames will be read by changing the line to read “for n = Span-9:Span”. Each frame was then converted to double precision format, permitting data to be stored as 64 bits. The pixel intensity was then normalized for each frame such that the minimum value was 0 and the maximum 255. To compute the magnitude of the gradient of the normalized intensity, central difference derivatives were used to compute the x and y components of the gradient. This routine utilized subroutine *Cshift2*, a circular shift routine for two-dimensional images, written by Charles Wolgemuth. This subroutine is included within the provided code for the ProcessDNA algorithm. A segmented image was then created using the squared magnitude of the gradient as a weighting factor to define a threshold for the image intensity. The segmented image is a binary array where pixels in the original image that have intensities greater than this threshold value are set to one and all other pixels are set to zero. The MATLAB *bwlabel* command was used to label connected regions in the segmented image. The MATLAB command *regionprops* was then used to determine the area, perimeter, eccentricity, major axis length, and minor axis length for each connected region. To compute the x and y coordinates of the center of mass for each nucleus, the integrated intensity and the Shape Factor (Shape = 4 π^2^ Area/Perimeter^2^) for all connected regions that exceed a specified area was determined. The center of mass, area, perimeter, integrated intensity, standard deviation of the integrated intensity, and shape factor for each region that exceeds the specified area were stored. The original image was then plotted, labeling each connected region (nucleus) with a number and its integrated intensity. To quickly assess a distribution of integrated intensities for each frame, a histogram plot was called.
