# Supplementary figures and images for: Measuring DNA content in live cells by fluorescence microscopy
Source: Cell Div. 2018 Sep 4;13:6. doi: 10.1186/s13008-018-0039-z (PMC6123973; doi:10.1186/s13008-018-0039-z)

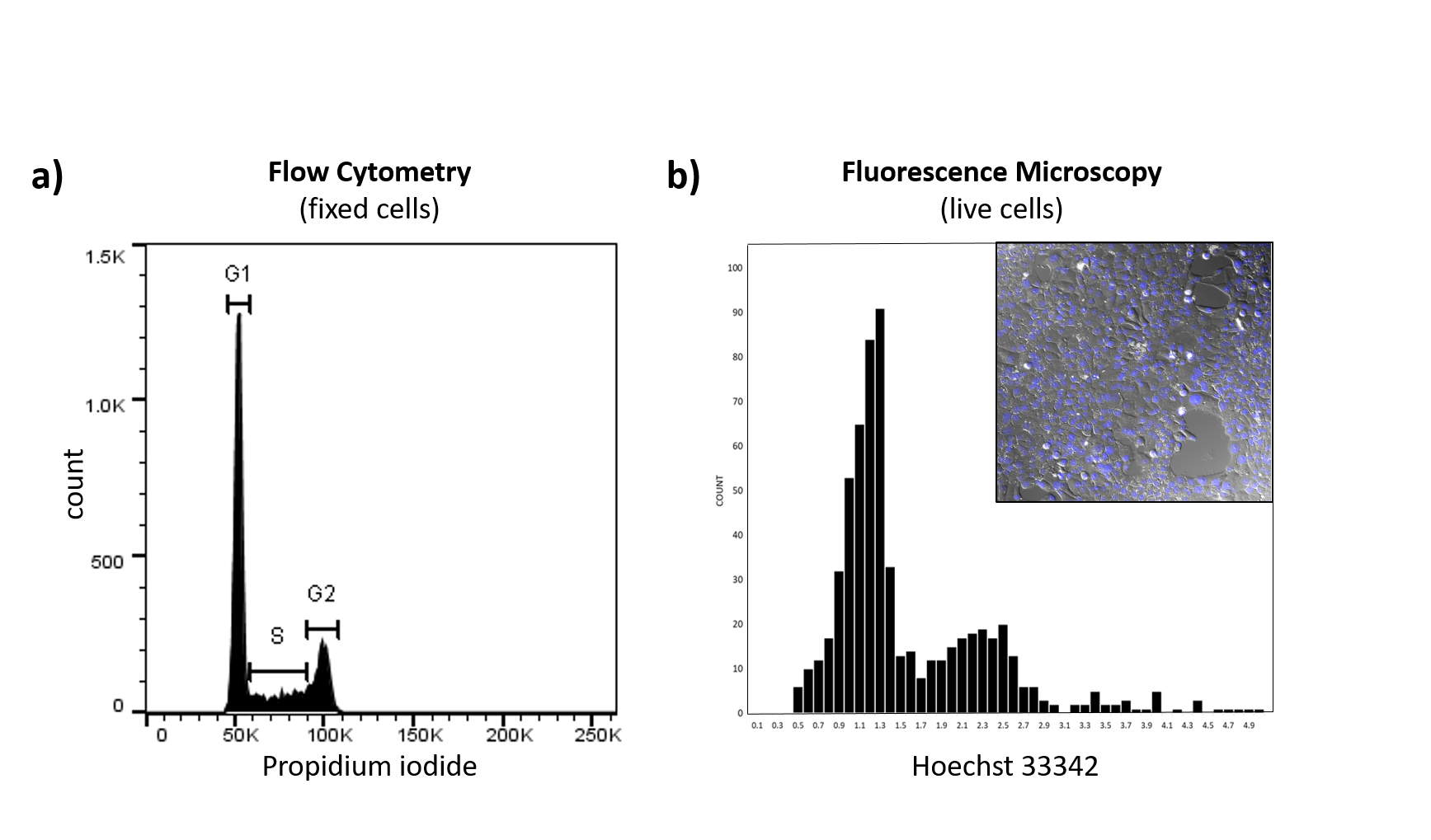

Supplement: Supplementary file 3 — Additional file 3: Figure S1. Side-by-side comparison of cell cycle profiles derived from flow cytometry and by live-cell fluorescence microscopy. Flow cytometry and fluorescence image analysis data was obtained from asynchronous cells that were plated in either 6-well culture dishes or 2-well chambered slides, respectively. a) The cell cycle profile displayed was generated from cells that were fixed and treated with propidium iodide and ribonuclease A. 10,000 events were collected with doublets gated out of the analysis by SCC and PI-A. Manual gates were placed to determine the percentage of cells within the different cell cycle phases shown. b) Displayed is a cell cycle profile generated from live-cell fluorescence microscopy of Hoechst 33342 stained cells (n = ~ 600 cells). The corresponding image is presented in the top-right corner to illustrate the plating density required for 600 cells per field of view. [file 13008_2018_39_MOESM3_ESM.tif]

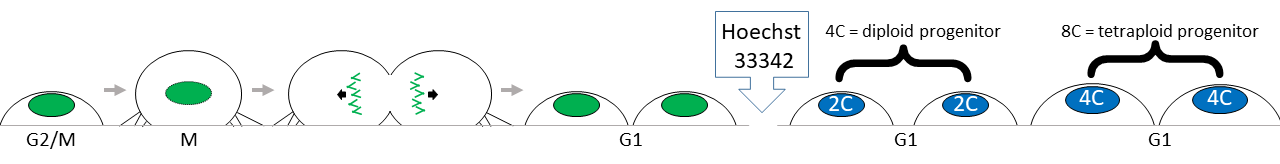

Supplement: Supplementary file 4 — Additional file 4: Figure S2. Calculating cellular ploidy in live cells using Hoechst 33342. A diagrammatical representation of H2B-GFP labeled cells progressing through mitosis (grey arrows) is shown. DNA ploidy can be calculated for each mitotic cell by summing the nuclear fluorescence of Hoechst 33342 in the nascent daughter cells. A diploid and tetraploid example is illustrated. [file 13008_2018_39_MOESM4_ESM.tif]
